# Management of Soft Tissue Sarcomas in Extremities: Variation in Treatment Recommendations and Surveillance According to Specialty and Continent

**DOI:** 10.1245/s10434-021-09946-4

**Published:** 2021-05-10

**Authors:** Ibtissam Acem, Merel M. Smit, Cornelis Verhoef, Winan J. van Houdt, Rick L. Haas, Jos A. van der Hage, Dirk J. Grünhagen, Michiel A. J. van de Sande

**Affiliations:** 1 Department of Surgical Oncology and Gastrointestinal Surgery, Erasmus MC Cancer Institute, Erasmus Medical Centre, Rotterdam, The Netherlands; 2 Department of Orthopedic Oncology, Leiden University Medical Centre, Leiden, The Netherlands; 3 Department of Surgical Oncology, The Netherlands Cancer Institute, Amsterdam, The Netherlands; 4 Department of Radiotherapy, The Netherlands Cancer Institute, Amsterdam, The Netherlands; 5 Department of Radiotherapy, Leiden University Medical Centre, Leiden, The Netherlands; 6 Department of Surgical Oncology, Leiden University Medical Centre, Leiden, The Netherlands

## Abstract

**Background:**

This study aimed to provide an insight into clinical decision-making and surveillance strategy of sarcoma specialists for patients with primary soft tissue sarcoma of the extremities (eSTS). The secondary aim was to quantify the role of patient- and tumor-specific factors in the perioperative management.

**Methods:**

Members of sarcoma societies were sent a Web-based 21-item survey about eSTS management. The survey concerned only primary resectable high-grade eSTS in adults.

**Results:**

The study enrolled 396 respondents. The majority of the surgical specialists thought the evidence for perioperative chemotherapy (CTX) for high-grade eSTS was insufficient. Radiotherapy (RTX) was less frequently offered in Asia than in North America and Europe. The specialties and continents also differed regarding the importance of patient and tumor characteristics influencing RTX and CTX recommendation. For surveillance after initial treatment outpatient visits, chest computed tomography (CT) scans, and magnetic resonance images of the extremity were the methods primarily used. The specialists in North America preferred chest CT scan over chest x-ray, whereas those in Asia and Europe had no clear preference.

**Discussion:**

Specialty and continent are important factors contributing to the variation in clinical practice, treatment recommendations, and surveillance of patients with primary resectable high-grade eSTS.

Soft tissue sarcomas (STSs) are a heterogeneous group of tumors with a mesenchymal origin. This group of malignant tumors has more than 80 histologic subtypes and accounts for 1% of all adult malignancies.^1^ Soft tissue sarcomas are rare, with an estimated incidence of about 5 patients per 100,000 persons in Europe every year.^2^,^3^ All this together makes it challenging to generate high-level evidence for the management of primary STS.

The National Comprehensive Cancer Network guideline (NCCN)^4^ and the European Society of Medical Oncology guideline (ESMO)^5^ are two broadly used international clinical practice guidelines for the management and surveillance of STS. The two guidelines are similar and agree that surgery is the cornerstone for the treatment of soft tissue sarcoma of the extremities (eSTS).^4^,^5^ Perioperative radiotherapy (RTX) is recommended to improve local control in settings wherein adequate margins are not possible or for high-grade, deep-seated tumors, or tumors 5 cm in size or larger.^4^,^5^ Perioperative chemotherapy (CTX) is not standard practice, but it can be offered as an option to high-risk patients after shared decision-making.^4^,^5^

Although several studies have shown that adherence to guidelines results in better patient outcomes, 32–70% of patients with STS are not consistently treated in accordance with the clinical guidelines.^6^–^11^ This study aimed to acquire insight into the variation of eSTS management by assessing the influence of clinical specialty and continent on clinical practice and surveillance. Additionally, this study investigated the extent to which selected patient and disease characteristics are used to distinguish between high- and low-risk patients and the extent to which these factors are used in clinical decision-making for perioperative treatment.

## Methods

### Survey Design

The survey used for this study was developed by the authors after literature review and a small focus group discussion. Pilot testing of the survey was performed internally for content and face validity at the Leiden University Medical Center, The Netherlands Cancer Institute, and the Erasmus MC Cancer Institute in The Netherlands. Online survey software (Qualtrics; Provo, UT, USA) was used to administer the survey, which was open to respondents for a 4-month period from 2 March to 2 July 2020.

The study population received an invitation e-mail from the participating sarcoma societies describing the purpose of the survey and containing an electronic link to the online survey software. The study population received two new invitations within a time frame of 4 months as a reminder. An opt-out option was provided in the request e-mail.

The survey included questions pertaining to respondent characteristics, the current clinical practice, the importance of selected patient and disease characteristics in the recommendation of perioperative treatment, and follow-up evaluation. Most of the questions required scoring of characteristics on a 5-point Likert scale. The survey was designed with closed-ended questions to allow a completion time of only about 10 min. The respondents were allowed to leave a question blank.

The 21-item survey is available in Appendix 1. The questions in the survey concerned only primary eSTS in adults (age ≥ 18 years). Additional treatment with isolated limb perfusion, immunotherapy, and regional hyperthermia were not considered in this survey.

The survey responses were anonymously collected, and no information that could potentially identify a respondent was collected. This study was approved by the institutional Medical Ethical Committee Leiden-Den Haag-Delft (N20.016) and complied with the regulations governing Good Clinical Research Practice and General Data Protection Regulation.

### Study Population

The target group for the questionnaire comprised clinically active international members of the Connective Tissue Oncology Society (CTOS), the European Musculo-Skeletal Oncology Society (EMSOS), and the Asia Pacific Musculoskeletal Tumor Society (APMSTS). Respondents who were not physicians or did not have a self-declared interest in STS were excluded from the study.

### Real-World Data

Findings on perioperative treatment in eSTS were compared with the real-world data of 6265 patients who had surgically treated primary high-grade eSTS (age ≥ 18 years) from 21 sarcoma centers. Details on this retrospective cohort were reported by Acem et al.^12^

### Statistical Analysis

All analyses were performed using the statistical program R (R Core Team, Vienna, Austria).^13^ Respondent characteristics and other categorical variables are described in absolute values and proportions. The 5-point Likert scale scores (5-pt LSS) were displayed in proportions and means (mean 5-point LSS) with standard deviations (SDs).

All the questions were stratified by specialty and continent. The respondents with a specialty in both medical and radiation oncology (clinical oncology) were classified as medical oncologists. The respondents from Africa, Central and South America, Australia, New Zealand, and Oceania were excluded from the analyses stratified by continent due to insufficiently large sample sizes.

Differences in outcomes on the 5-pt LSS were tested with the one-way analysis of variance (ANOVA) test. Differences in categorical outcomes were tested with the chi-square test or Fisher’s exact test when the value of at least one cell in the contingency table was below 5. Bonferroni correction was used to account for multiple testing. Blank questions were considered missing and not imputed.

## Results

### Demographics

The survey was received by 1386 potential respondents and completed by 428 respondents (response rate, 30.9%), 396 of whom met the inclusion criteria. The study excluded respondents without a special interest in STS (*n* = 31) and respondents who were not physicians (*n* = 1). The last question of the survey was answered by 255 respondents (64.4%). Appendix 2 presents a flowchart of the respondent inclusion.

The baseline characteristics of the respondents are depicted in Table 1. Most of the respondents were orthopedic oncologists (43.2%, *n* = 171) practiced in Europe (44.9%, *n* = 155) and had more than 15 years of experience after their fellowship (36.9%, *n* = 146).

**Table 1:** Baseline characteristics of the respondents

Characteristics	Overall (*n* = 396) *n* (%)
*Specialty*	
Medical oncology	89 (22.5)
Orthopedic oncology	171 (43.2)
Radiation oncology	28 (7.1)
Surgical oncology	83 (21.0)
Other^a^	25 (6.3)
*Years since completion of fellowship*	
I am a fellow in training	15 (3.8)
< 5 years	73 (18.5)
5–10 years	90 (22.8)
11–15 years	65 (16.5)
≥ 15 years	151 (38.3)
Missing	2
*Current practice location*	
Africa	3 (0.8)
Asia	83 (21.0)
Australia/New Zealand/Oceania	16 (4.0)
Central/South America	7 (1.8)
Europe	155 (39.1)
North America	132 (33.3)
*Number of new cases annually*	
< 5	28 (7.1)
5–25	95 (24.0)
25–50	92 (23.2)
≥ 50	181 (45.7)

^a^Including pediatric and adolescent oncology and pathology

### Distinction Between High- and Low-Risk Patients

**Fig. 1:**
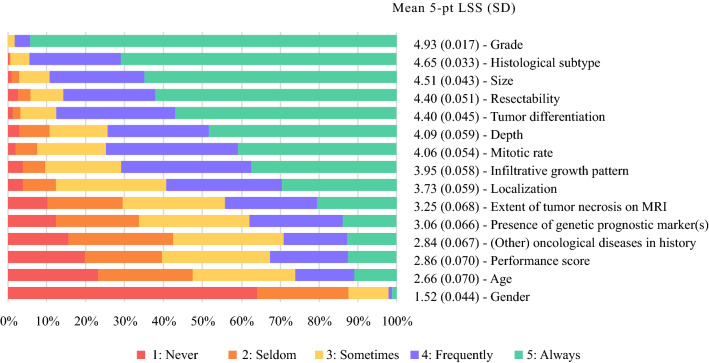
Use of patient and disease characteristics to distinguish between high- and low-risk patients with soft tissue sarcoma of the extremity (eSTS) (*n* = 348).* 5-pt LSS* 5-point Likert scale score, *SD* standard deviation

The characteristics primarily used to distinguish between high- and low-risk eSTS patients were grade (mean 5-pt LSS, 4.93), histologic subtype (mean 5-pt LSS, 4.65), and size (mean 5-pt LSS, 4.51) (Fig. 1). Gender (mean 5-pt LSS, 1.52) and age (mean 5-pt LSS, 2.66) were the least important factors used to distinguish between high- and low-risk eSTS patients.

For surgical specialties, extent of tumor necrosis on MRI (mean 5-pt LSS, 3.51) and infiltrative growth pattern (mean 5-pt LSS, 4.12) were more important for distinguishing between high- and low-risk patients than non-surgical specialties (mean 5-pt LSS, 3.51 vs 2.84 [*p* < 0.001] and 4.12 vs 3.56 [*p* < 0.001], respectively). For non-surgical specialties, size (mean 5-pt LSS, 4.75) was more important for distinguishing between high- and low-risk patients than surgical specialties (mean 5-pt LSS, 4.40; *p* < 0.001). The use of patient and disease characteristics stratified by specialty are depicted in Appendix 3.

To distinguish between high- and low-risk patients, the specialists in Asia and Europe gave a higher rating of importance than the specialists in North America for extent of tumor necrosis on MRI (mean 5-pt LSS, 3.75 vs 2.80 [*p* < 0.001] and 3.43 vs 2.80 [*p* < 0.001], respectively) and infiltrative growth pattern (mean 5-pt LSS, 4.21 vs 3.71 [*p* < 0.001] and 4.11 vs 3.71 [*p* = 0.004], respectively).

### Current Practice of RTX in the Management of High-Grade eSTS

**Fig. 2:**
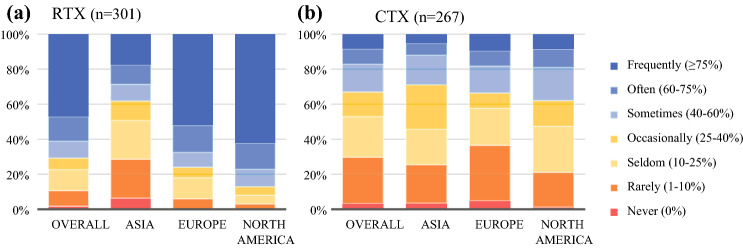
What percentage of your patients with high-grade soft tissue sarcoma of the extremity (eSTS) receive perioperative treatment? **a** Radiotherapy. **b** Chemotherapy.

Of the 301 respondents, 142 (47.2%) treated their high-risk eSTS patients frequently (≥75%) with perioperative RTX. In Asia, RTX was offered less often (17.5%) than in Europe (52.1%; *p* < 0.001) or North America (62.4%; *p* < 0.001) (Fig. 2a). This was in accordance with the real-world data showing that 19.6% of the patients received RTX in Asia compared with 62.2% in Europe (*p* < 0.001) and 74.3% in Europe and North America (*p* < 0.001) (Appendix 4).

### Factors Influencing RTX Recommendation

**Fig. 3:**
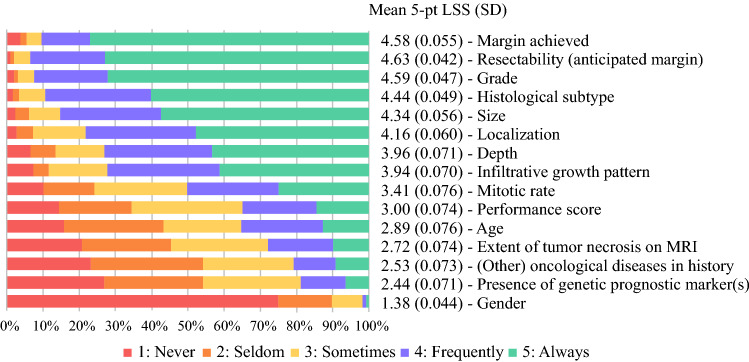
Factors influencing radiotherapy (RTX) recommendation (*n* = 291). 5-pt LSS, 5-point Likert scale score. SD, standard deviation.

The factors most likely to influence perioperative RTX recommendation were the margins achieved (mean 5-pt LSS, 4.58), the anticipated margins (mean 5-pt LSS, 4.63), and grade (mean 5-pt LSS, 4.59) (Fig. 3). The least important factors influencing RTX recommendation were gender (mean 5-pt LSS, 1.38) and presence of a genetic prognostic markers (mean 5-pt LSS, 2.44).

For surgical specialties, infiltrative growth pattern was a more important factor influencing RTX recommendation (mean 5-pt LSS, 4.13) than nonsurgical specialties (mean 5-pt LSS, 3.51; *p* < 0.001). For nonsurgical specialties, grade (mean 5-pt LSS, 4.74), performance score (mean 5-pt  LSS, 3.33), and oncologic history (mean 5-pt LSS, 2.82) were more important factors influencing RTX recommendation than surgical specialties (mean 5-pt LSS: 4.52 [*p* = 0.025], 2.87 [*p* = 0.005], and 2.35 [*p* = 0.004], respectively). The use of patient and disease characteristics for RTX recommendation stratified by specialty are depicted in Appendix 5.

The specialists in Europe and North America rated grade for recommendation of perioperative RTX as more important than did the specialists in Asia (mean 5-pt LSS, 4.69 vs 4.22 [*p* = 0.003] and 4.83 vs 4.22 [*p* < 0.001], respectively). The specialists in North America rated size for the recommendation of perioperative RTX as more important than did the specialists in Asia and Europe (mean 5-pt LSS, 4.59 vs. 4.12 [*p* = 0.001] and 4.59 vs 4.29 [*p* = 0.020], respectively).

### Use of a Prediction Tool for RTX Recommendation

Of the 296 respondents 219 (74%) would consider using a prediction tool to indicate perioperative RTX for eSTS patients. Surgical oncologists (92.2%) would consider using a prediction tool more often than orthopedic oncologists (65.7%; *p* < 0.001). Specialists in Asia were less likely to consider using a prediction tool (50%) than specialists in Europe (76.1%; *p* < 0.001) or North America (84.2%; *p* < 0.001).

### Current Practice of CTX in the Management of High-Grade eSTS

Of the 276 respondents, 194 (70.3%) treated more than 10% of their high-risk eSTS patients with perioperative CTX (Fig. 2b). No significant differences were found among continents in the use of CTX for high-grade eSTS. However, the real-world data showed a significant difference in the use of CTX among continents. In Asia, CTX was administered to 30.6% of the patients, whereas perioperative CTX was administered to 12.6% of the patients in Europe (*p* < 0.001) and to 3.3% of the patients North America (*p* < 0.001) (Appendix 4).

Of the 276 respondents, 173 (62.7%) did not think the evidence was sufficient to use of perioperative CTX for patients with primary high-grade eSTS. The majority of the orthopedic (74%) and surgical (73.3%) oncologists (*p* < 0.001) considered the current level of evidence for the role of CTX in high-grade eSTS to be insufficient, compared with 35.7% of the medical oncologists (*p* < 0.001). The attitude toward the role of perioperative CTX in primary high-grade eSTS did not differ across continents (*p* = 0.137).

Older age (≥ 70 years) was thought by 120 (43%) of the 278 respondents to be an absolute contraindication for perioperative CTX.

### Factors Influencing CTX Recommendation

**Fig. 4:**
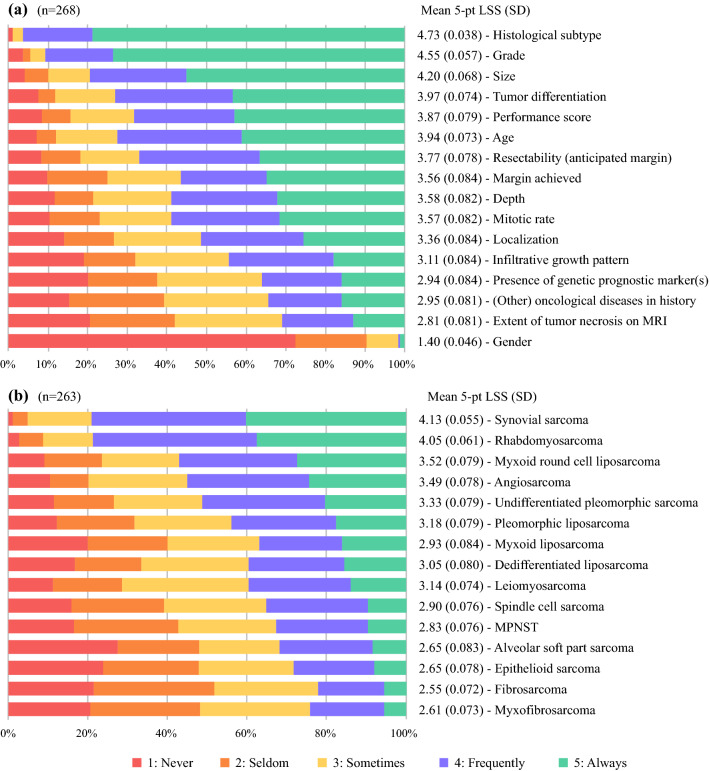
**a** Factors influencing chemotherapy (CTX) recommendation. **b** For which histologic subtypes would you generally consider perioperative chemotherapy (CTX)? 5-pt LSS, 5-point Likert scale score; SD, standard deviation; MPNST, malignant peripheral nerve sheath tumor

The factors most likely to influence perioperative CTX recommendation were histologic subtype (mean 5-pt LSS, 4.73), grade (mean 5-pt LSS, 4.55), and size (mean 5-pt LSS, 4.20). The least important factors influencing CTX recommendation were gender (mean 5-pt LSS, 1.40) and extent of tumor necrosis on MRI (mean 5-pt LSS, 2.81) (Fig. 4a).

For the nonsurgical specialties, depth (mean 5-pt LSS, 3.92), location (mean 5-pt LSS, 3.62), performance score (mean 5-pt LSS, 4.36), and size (mean 5-pt LSS, 4.57) were more important factors influencing CTX recommendation than for the surgical specialties (mean 5-pt LSS, 3.41 [*p* = 0.004], 3.20 [*p* = 0.020], 3.64 [*p* < 0.001], 3.98 [*p* < 0.001], respectively). The use of patient and disease characteristics for CTX recommendation stratified by specialty are depicted in Appendix 6.

The specialists in Asia and Europe compared with the specialists in North America gave a higher rate of importance to extent of tumor necrosis on MRI (mean 5-pt LSS, 3.13 vs 2.30 [*p* < 0.001] and 3.08 vs 2.30 [*p* < 0.001], respectively) and infiltrative growth pattern (mean 5-pt LSS, 3.36 vs 2.76 [*p* = 0.005] and 3.30 vs. 2.76 [*p* = 0.003], respectively) for a perioperative CTX recommendation.

The respondents would consider perioperative CTX primarily for synovial sarcoma (mean 5-pt LSS, 4.13), rhabdomyosarcoma (mean 5-pt LSS, 4.05), and myxoid liposarcoma with a round cell component (mean 5-pt LSS, 3.52). Perioperative CTX would be considered the least for fibrosarcoma (mean 5-pt LSS, 2.55) and myxofibrosarcoma (mean 5-pt LSS, 2.61) (Fig. 4b).

### Use of a Prediction Tool for CTX Recommendation

Of the 277 respondents, 224 (80.9%) would consider using a prediction tool to indicate perioperative CTX for eSTS patients. The specialists did not differ significantly in their attitude toward using a prediction tool for CTXs. The surgical oncologists (92.2%) would consider using a prediction tool more often than the orthopedic oncologists (65.7%; *p* < 0.001). The specialists in Asia were less likely to consider using a prediction tool (62.7%) than the specialists in Europe (82.9%; *p* = 0.007) or North America (88.4%; *p* < 0.001).

### Follow-up Evaluation

Outpatient visits, chest CT scan, and MRI of the extremity were the most common methods for follow-up evaluation. The frequency of each method declined with time (Table 2). The specialists in North America preferred chest CT scan over chest x-ray, with a median of four chest CT scans (mean, 3.33) in the first year compared with no chest x-rays (mean, 0.860) (*p* < 0.001). After the first year, chest CT scan remained the preferred method in North America. Neither of the two methods were clearly preferred by specialists in Asia (median for CT vs. x-ray in the first year, 2 vs. 3; *p* = 0.276) or Europe (median for CT vs. x-ray in the first year, 2 vs. 2; *p* = 0.520). In the first 5 years of surveillance, 29% of the respondents never used chest x-ray, and 12% of the respondents never used chest CT scan. The outpatient clinic visit sequence used primarily in the first 5 years was 4-4-2-2-2 (16.9%; 42 of 248).

**Table 2:** Follow-up schedule per year after initial treatment for high-grade soft tissue sarcoma of the extremity (eSTS) (*n* = 252)

Method	Mean no. per year (median)				
	Year 1	Year 2	Year 3	Year 4	Year 5
Outpatient visit	3.94 (4)	3.47 (4)	2.66 (2)	2.43 (2)	2.29 (2)
Chest x-ray	1.89 (2)	1.82 (2)	1.49 (1)	1.39 (1)	1.27 (1)
Chest CT	2.65 (3)	2.48 (3)	1.95 (2)	1.70 (1)	1.48 (1)
Extremity x-ray	1.17 (0)	0.968 (0)	0.807 (0)	0.892 (0)	0.743 (0)
Extremity CT	0.565 (0)	0.591 (0)	0.489 (0)	0.525 (0)	0.397 (0)
Extremity MRI	2.55 (3)	2.43 (2)	1.94 (2)	1.86 (1)	1.65 (1)
PET-CT scan	0.667 (0)	0.510 (0)	0.384 (0)	0.358 (0)	0.476 (0)

*CT* computed tomography, *MRI* magnetic resonance imaging, *PET* positron emission tomography

**Fig. 5:**
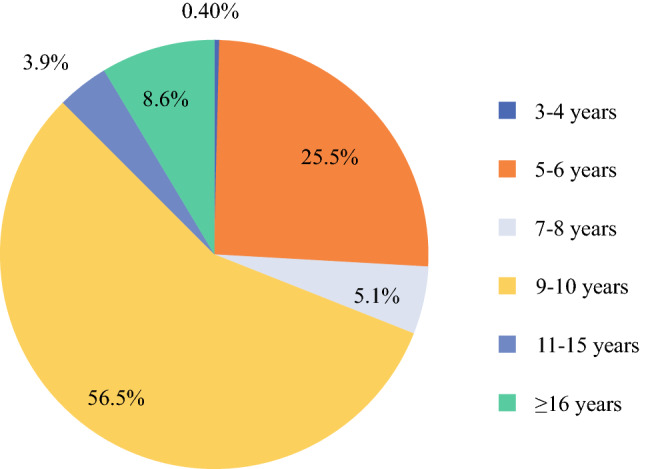
Duration of follow-up period after primary treatment (*n* = 255)

Most of the respondents (56.9%) felt comfortable to end the surveillance in patients with primary high-grade eSTS after 9 to 10 years of follow-up evaluation. Whereas 8.6% would follow their patients for more than 16 years or for their whole lifetime, 26% of the respondents ended the surveillance after 5 to 6 years (Fig. 5)

### Discussion

This study aimed to provide an insight into variation in the clinical decision-making processes between specialties and continents for the treatment of resectable high-grade eSTS. In addition, it aimed to analyze the relative role of specific tumor and patient factors in the clinical decision-making with regard to the perioperative treatment of these patients. This study illustrates a wide variation among specialties and continents regarding the management and surveillance of patients with eSTS. Also, the results indicate a variation in risk factors considered to be indications for perioperative treatment. However, consensus exists regarding the risk factors frequently leading to recommendation for RTX (margins, grade, histologic subtype, size) and CTX (size, histologic subtype, grade).

This study demonstrated a notable difference in RTX practice among continents, in accordance with the included real-world data.^12^ In Europe and North America, most of the respondents treat 75% or more of their patients with high-grade resectable eSTS using perioperative RTX, compared with only 17.5% of the respondents in Asia. Also, we observed a greater variation of RTX use in Asia than in Europe and North America. These results are supported by a systematic review including 24 studies of the Asia-Pacific region in which the use of RTX ranged from 1 to 100% preoperatively and from 6 to 88% postoperatively.^14^ The on-average lower rates of RTX use and greater variation of RTX use in Asia might be explained by a generally lower accessibility to radiotherapy in certain Asia-Pacific regions.^15^

The survey did not demonstrate a difference in CTX practice among continents. However, a notable difference in CTX use among the continents was observed in the real-world data, with CTX administration more prevalent in Asia than in Europe or North America.^12^ However, the real-world data included only one high-volume center from North America and only Japanese centers from Asia.^12^

The attitude toward the role of CTX in the management of eSTS varies widely. More than 70% of the orthopedic and surgical oncologists did not think the evidence is sufficient for CTX in primary high-grade resectable eSTS, compared with 35% of the medical oncologists. Substantial variation also exists in the current practice of perioperative chemotherapy, with 30% of the respondents never or rarely using CTX, but with almost half of the respondents (47%) using perioperative CTX for more than 25% of their patients with primary high-grade eSTS. The variation in CTX practice might reflect a difference in interpretation of the available evidence on the role of perioperative CTX in primary eSTS. Other factors that might explain the variation are the availability of perioperative treatment and the variety of compensation and health care systems.

Several studies have suggested that a selected group of high-risk patients might benefit from perioperative CTX.^16^,^17^ However, the identification of these high-risk patients remains challenging. Our study demonstrated that the most important factors physicians use to identify high-risk patients are grade, histologic subtype, and size. These factors also are included in prediction tools such as the Sarculator (Callegaro et al., Milan, Italy) and PERSARC (Van Praag et al., Leiden, The Netherlands).^18^,^19^ The respondents of this study were predominantly positive about using such prediction tools to select patients for perioperative treatment. Interestingly, genetic prognostic markers are less widely used in the identification of high-risk patients, whereas genetic prognostic markers seem promising for the identification of high-risk patients. Chibon et al.^20^ showed that the gene expression profile, CINSARC, was a strong independent predictor for progressive disease and might identify high-risk patients that could benefit from CTX.^21^,^22^

Physicians seem to use different factors as indicators for RTX compared with CTX, which makes sense considering that RTX aims to improve local control, whereas CTX aims to prevent distant disease. Surgical margins play an important role in the indication for RTX, as shown by Wasif et al.^23^ In contrast, the most important factor in the indication for CTX is histologic subtype. Physicians would consider perioperative CTX the most frequently for synovial sarcoma and rhabdomyosarcoma. The importance of using these factors in the indication for RTX and CTX provides an interesting insight into the clinical decision-making process of physicians. This could be helpful for future studies because it quantifies the importance of adjusting for these factors in any observational study analyzing the role of perioperative treatment.

The variation in administration of perioperative treatment among specialties and continents might arise from the lack of available evidence on eSTS management that may be sufficient to standardize clinical decision-making. The rarity of eSTS makes it challenging to conduct well-powered trials of perioperative treatment. Also, the multiple biologic subtypes, anatomic variability, and limited understanding of tumor biology and the tumor immune microenvironment of multiple subtypes impose difficulties on clinical trial design compared with clinical trials of perioperative treatment for other more prevalent cancers with more homogeneous populations. However, the variation in perioperative treatment also might arise from less knowledge of the literature outside a practitioner’s clinical domain.^23^,^24^ In addition, the clinical guidelines leave room for interpretation and variation.^4^,^5^ These factors reflect the importance of a multidisciplinary expert board reaching consensus decisions and facilitating personalized sarcoma care.

Only a few studies have investigated the optimal routine follow-up policy for patients with localized high-grade eSTS.^25^,^26^ Therefore, the optimal frequency and intensity of the routine follow-up policy remains unclear. The current clinical guidelines recommend follow-up evaluation every 3 to 4 months in the first 2 to 3 years, then twice a year up to the fifth year and once a year thereafter.^4^,^5^ The guidelines do not specify whether chest CT scan or chest x-ray should be used in the follow-up evaluation. This study showed that physicians in North America have a clear preference for chest CT scan over chest x-ray, whereas in Asia and Europe, no preference between these methods was found. The variability of follow-up strategies found in this study and other studies demonstrates the urgent need for well-designed prospective studies on follow-up evaluation.^27^–^30^

This study had some limitations. Only closed-ended questions were used to minimize the completion time and to maximize the completion rate. This resulted in a simplification of the responses. To prevent a lack of depth in the questionnaire and to prevent question order bias, a broad range of answers were included and arranged alphabetically. We recognize that other variables not captured in the questionnaire may also influence the choice for perioperative treatment.

Additionally, the use of a survey had the inherent limitation of selection bias because only physicians inclined to respond took time to do so. Also, the survey was sent only to active members of selected sarcoma societies, with some continents and specialties underrepresented in this study, which might affect the generalizability of our results.

Although electronic dissemination of the survey enabled easy delivery and reply, many e-mail addresses were invalid, and many e-mails were bounced back from e-mail filters. This might partially explain our moderate response rate of 31%. The high response rate (79%) of those who did open the e-mail shows that once the e-mail was received by the respondents, most of them went on complete the survey.

In conclusion, although several studies have shown that adherence to clinical guidelines results in better patient outcomes, this study showed remarkable variation in the management of eSTS. Specialty and continent are important factors contributing to the variation in clinical practice, treatment recommendations, and surveillance of patients with primary resectable high-grade eSTS.

## Appendix 1: Survey

### What is the questionnaire about?

To investigate the variation in treatment policies regarding localized high grade (grade 2–3) soft tissue sarcomas of extremities (eSTS) and to get a better understanding of important patient and disease characteristics influencing disease management we would like to invite you to fill in this questionnaire.

The questionnaire consists of 20 questions. You can save the questionnaire at any time and complete it later. The questionnaire takes about 10 minutes to complete.

The questions in this questionnaire concern only primary (non-metastasized) high grade soft tissue sarcomas of extremities in adults (≥ 18 years). Additional treatment with isolated limb perfusion (ILP), immunotherapy and regional hyperthermia (RH) are not considered in this questionnaire.

### Respondent characteristics

1. Are you a physician with an interest in soft tissue sarcomas? Yes/no

2. What is your speciality?

a. Medical oncology

b. Orthopaedic oncology

c. Radiation oncology

d. Surgical oncology

e. Other

3. How many years have elapsed since completion of your fellowship?

a. I am a fellow in-training

b. < 5 years

c. 5–10 years

d. 11–15 years

e. > 15 years

f. Other

4. Where do you practice?

a. Africa

b. Asia

c. Australia/New Zealand/Oceania

d. Central/South America

e. Europe

f. North America

5. How many new cases of extremity soft tissue sarcoma do you treat in your hospital annually? (average experience over the last 5 years):

a. < 5 per year

b. 5–25 per year

c. 25–50 per year

d. > 50 per year

## STS management

### General

6. Which patient and/or disease characteristics do you use to distinguish between high-risk and low-risk STS patients on a scale of 1-5 (1: never, 5: always)?

a. Age

b. Depth

c. Extent of tumour necrosis on MRI

d. Gender

e. Grade

f. Histological subtype

g. Infiltrative growth pattern

h. Localization

i. Mitotic rate

j. Performance score (WHO/KPS)

k. Presence of genetic prognostic marker(s)

l. Presence of (other) oncological diseases in history

m. Resectability: anticipated margin R0-R1-R2

n. Size

o. Tumour differentiation

### Radiotherapy

7. What percentage of your patients with high-grade (grade 2–3) primary eSTS receive (neo)adjuvant radiotherapy? (scroll bar)

8. Which of the following patient and/or disease characteristics do generally influence your choice for treatment with (neo)adjuvant radiotherapy on a scale of 1–5 (1: never, 5: always)?

a. Age

b. Depth

c. Extent of tumour necrosis on MRI

d. Gender

e. Grade

f. Histological subtype

g. Infiltrative growth pattern

h. Localization

i. Margin achieved: R0-R1-R2

j. Mitotic rate

k. Performance score (WHO/KPS)

l. Presence of genetic prognostic marker(s)

m. Presence of (other) oncological diseases in history

n. Resectability: anticipated margin R0-R1-R2

o. Size

p. Tumour differentiation

9. When making decisions regarding radiotherapy in addition to surgery in patients with primary eSTS, at what cut-off value of the predicted 5-year local recurrence rate would you recommend (neo)adjuvant radiotherapy? (scroll bar)*

10. When making decisions regarding radiotherapy in addition to surgery in patients with primary eSTS, at what cut-off value of the absolute 5-year local recurrence rate reduction (ARR) would you recommend (neo)adjuvant radiotherapy? (scroll bar)*

## Would you consider using a prediction tool for local recurrence, such as Sarculator or Persarc, to indicate (neo)adjuvant radiotherapy in eSTS patients? Yes/No

### Chemotherapy

12. What percentage of your patients with high-grade (grade 2–3) primary eSTS receive (neo)adjuvant chemotherapy? (scroll bar)

13. Which of the following patient and/or disease characteristics do generally influence your choice for treatment with (neo)adjuvant chemotherapy on a scale of 1–5 (1 = never, 5 = always)?

a. Age

b. Depth

c. Extent of tumour necrosis on MRI

d. Gender

e. Grade

f. Histological subtype

g. Infiltrative growth pattern

h. Localization

i. Margin achieved: R0-R1-R2

j. Mitotic rate

k. Performance score (WHO/KPS)

l. Presence of genetic prognostic marker(s)

m. Presence of (other) oncological diseases in history

n. Resectability: anticipated margin R0-R1-R2

o. Size

p. Tumour differentiation

14. For what predicted 5-year mortality risk do you consider (neo)adjuvant chemotherapy in addition to surgery in primary eSTS? (scroll bar)*

15. For what absolute 5-year mortality risk reduction (ARR) do you consider chemotherapy in addition to surgery in primary eSTS? (scroll bar)*

16. Do you feel there is sufficient evidence to use (neo)adjuvant chemotherapy for treatment of primary high-grade (grade 2–3) resectable eSTS? Yes/No

17. Would you consider using a prediction tool for overall survival and/or distant metastasis risk, such as Sarculator or Persarc, to indicate (neo)adjuvant chemotherapy in eSTS patients? Yes/No

18. In which STS histologic subtypes (grade 2/3, deep-seated, > 5 cm) would you generally consider (neo)adjuvant chemotherapy on a scale of 1–5 (1= never, 5= always)?

a. Alveolar soft part sarcoma

b. Angiosarcoma

c. Dedifferentiated liposarcoma

d. Epithelioid sarcoma

e. Fibrosarcoma

f. Leiomyosarcoma

g. Malignant peripheral nerve sheath tumour (MPNST)

h. Myxofibrosarcoma

i. Myxoid liposarcoma

j. Pleomorphic liposarcoma

k. Rhabdomyosarcoma

l. Round cell liposarcoma

m. Spindle cell sarcoma

n. Synovial sarcoma

o. Undifferentiated sarcoma

## Would older age (> 70 years) be an absolute contra-indication for (neo)adjuvant chemotherapy? Yes/No

### Follow-up

20. What is your follow-up schedule after initial treatment (with surgery and (neo)adjuvant treatment if indicated) has been completed for primary high-grade eSTS? Please enter the number of times during each time interval.

**Table:**

	1st year	2nd year	3rd year	4th year	5th year
Outpatient clinic visit					
X-thorax					
CT thorax					
X-extremity					
CT extremity					
MRI extremity					
PET-CT scan					

21. After how many years of disease free survival would you feel comfortable to end the follow-up of your patient with primary high-grade eSTS?

*The results of question 9, 10, 14, 15 have not been reported, as these questions were interpreted in multiple ways. Therefore, the results were not reliable.

## Appendix 3: The use of patient and disease characteristics to distinguish between high-risk and low-risk eSTS patients stratified by specialty

**Table:**

	Mean 5-point Likert Scale Score (Standard error of the mean)					
	Overall	Medical oncology	Orthopaedic oncology	Radiation oncology	Surgical oncology	*p**
Age	2.66 (0.070)	2.61 (0.147)	2.69 (0.098)	2.08 (0.223)	2.83 (0.168)	0.090
Depth	4.09 (0.059)	4.31 (0.106)	4.18 (0.080)	3.64 (0.336)	3.84 (0.136)	**0.007**
Extent of tumor necrosis on MRI	3.25 (0.068)	2.88 (0.143)	3.53 (0.096)	2.71 (0.244)	3.45 (0.147)	**0.000**
Gender	1.52 (0.044)	1.37 (0.077)	1.54 (0.070)	1.33 (0.130)	1.72 (0.105)	**0.044**
Grade	4.93 (0.017)	4.93 (0.036)	4.91 (0.029)	5.00 (0.000)	4.92 (0.035)	0.602
Histological subtype	4.65 (0.033)	4.73 (0.056)	4.59 (0.051)	4.38 (0.189)	4.70 (0.068)	0.052
Infiltrative growth pattern	3.95 (0.058)	3.50 (0.117)	4.15 (0.079)	3.79 (0.225)	4.07 (0.136)	**0.000**
Localization	3.73 (0.059)	3.77 (0.108)	3.78 (0.086)	3.64 (0.233)	3.50 (0.160)	0.370
Mitotic rate	4.06 (0.054)	4.11 (0.103)	4.07 (0.079)	3.54 (0.233)	4.20 (0.121)	**0.046**
Performance score	2.86 (0.070)	3.00 (0.157)	2.84 (0.098)	2.83 (0.274)	2.88 (0.162)	0.833
Presence of genetic prognostic maker(s)	3.06 (0.066)	3.02 (0.148)	3.13 (0.094)	2.75 (0.250)	2.95 (0.151)	0.478
Presence of (other) oncological diseases in history	2.84 (0.067)	2.65 (0.142)	2.97 (0.095)	2.79 (0.282)	2.67 (0.153)	0.184
Resectability	4.40 (0.051)	4.43 (0.101)	4.45 (0.074)	4.46 (0.217)	4.20 (0.123)	0.337
Size	4.51 (0.043)	4.81 (0.059)	4.52 (0.056)	4.56 (0.164)	4.10 (0.132)	**0.000**
Tumor differentiation	4.40 (0.045)	4.20 (0.118)	4.46 (0.059)	4.40 (0.173)	4.51 (0.076)	0.088

*Global *p* value for difference in distribution among specialties.

## Appendix 4: Perioperative therapy in a cohort of high-grade eSTS patients

**Table:**

	Overall (*N* = 6260)	Asia (*N* = 1850)	Europe (*N* = 3304)	North America^#^ (*N* = 1106)	*p**
Surgical margin					< 0.001
R0	5338 (85.3%)	1764 (95.4%)	2630 (79.6%)	944 (85.4%)	
R1-R2	732 (11.7%)	86 (4.6%)	484 (14.6%)	162 (14.6%)	
*Missing*	*190 (3.0%)*	*0 (0%)*	*190 (5.8%)*	*0 (0%)*	
*Radiotherapy*					< 0.001
0	3016 (48.2%)	1488 (80.4%)	1247 (37.7%)	281 (25.4%)	
1	3239 (51.7%)	362 (19.6%)	2055 (62.2%)	822 (74.3%)	
*Missing*	*5 (0.1%)*	*0 (0%)*	*2 (0.1%)*	*3 (0.3%)*	
*Chemotherapy*					< 0.001
0	5240 (83.7%)	1283 (69.4%)	2889 (87.4%)	1068 (96.6%)	
1	1019 (16.3%)	567 (30.6%)	415 (12.6%)	37 (3.3%)	
*Missing*	*1 (0.0%)*	*0 (0%)*	*0 (0%)*	*1 (0.1%)*	

*Global *p* value for difference in distribution among continents.

^#^Only data of one centre in North America was available.

## Appendix 5: Patient and disease characteristics influencing RTX recommendation stratified by specialty

**Table:**

	Mean 5-point Likert Scale Score (Standard error of the mean)					
	Overall	Medical oncology	Orthopaedic oncology	Radiation oncology	Surgical oncology	*p**
Age	2.89 (0.076)	2.79 (0.158)	2.91 (0.117)	2.95 (0.270)	2.82 (0.139)	0.912
Depth	3.96 (0.071)	4.20 (0.139)	3.94 (0.105)	3.95 (0.301)	3.78 (0.157)	0.270
Extent of tumor necrosis on MRI	2.72 (0.074)	2.70 (0.164)	2.65 (0.111)	2.74 (0.274)	2.90 (0.152)	0.710
Gender	1.38 (0.044)	1.26 (0.073)	1.38 (0.065)	1.21 (0.123)	1.58 (0.131)	0.097
Grade	4.59 (0.047)	4.77 (0.083)	4.46 (0.078)	4.65 (0.209)	4.69 (0.066)	**0.046**
Histological subtype	4.44 (0.049)	4.47 (0.103)	4.36 (0.076)	4.30 (0.206)	4.65 (0.078)	0.178
Infiltrative growth pattern	3.94 (0.070)	3.39 (0.167)	4.20 (0.090)	3.95 (0.270)	3.98 (0.155)	**0.000**
Localization	4.16 (0.060)	4.21 (0.123)	4.17 (0.087)	4.20 (0.156)	4.02 (0.160)	0.774
Margin achieved	4.58 (0.055)	4.62 (0.113)	4.52 (0.090)	4.60 (0.210)	4.67 (0.078)	0.785
Mitotic rate	3.41 (0.076)	3.57 (0.164)	3.22 (0.112)	2.95 (0.259)	3.82 (0.153)	**0.008**
Performance score	3.00 (0.074)	3.33 (0.157)	2.70 (0.105)	3.32 (0.230)	3.34 (0.166)	**0.001**
Presence of genetic prognostic maker(s)	2.44 (0.071)	2.46 (0.154)	2.35 (0.098)	2.32 (0.254)	2.68 (0.182)	0.382
Presence of (other) oncological diseases in history	2.53 (0.073)	2.82 (0.173)	2.28 (0.100)	2.84 (0.245)	2.54 (0.146)	**0.013**
Resectability	4.63 (0.042)	4.71 (0.093)	4.61 (0.060)	4.45 (0.223)	4.65 (0.073)	0.526
Size	4.34 (0.056)	4.60 (0.107)	4.26 (0.085)	4.35 (0.221)	4.20 (0.122)	0.063
Tumor differentiation	3.99 (0.067)	3.97 (0.156)	3.88 (0.099)	3.70 (0.282)	4.37 (0.093)	**0.039**

*Global *p* value for difference in distribution among specialties.

## Appendix 6: Patient and disease characteristics influencing CTX recommendation stratified by specialty

**Table:**

	Mean 5-point Likert Scale Score (Standard error of the mean)					
	Overall	Medical oncology	Orthopaedic oncology	Radiation oncology	Surgical oncology	*p**
Age	3.94 (0.073)	4.07 (0.136)	4.02 (0.106)	4.26 (0.263)	3.54 (0.171)	0.055
Depth	3.58 (0.082)	3.97 (0.158)	3.60 (0.116)	3.72 (0.351)	2.83 (0.188)	**0.000**
Extent of tumor necrosis on MRI	2.81 (0.081)	2.67 (0.165)	3.02 (0.115)	2.39 (0.315)	2.71 (0.188)	0.112
Gender	1.40 (0.046)	1.38 (0.098)	1.43 (0.066)	1.22 (0.129)	1.39 (0.120)	0.752
Grade	4.55 (0.057)	4.69 (0.114)	4.50 (0.081)	4.63 (0.232)	4.41 (0.153)	0.394
Histological subtype	4.73 (0.038)	4.76 (0.087)	4.70 (0.050)	4.83 (0.090)	4.69 (0.110)	0.803
Infiltrative growth pattern	3.11 (0.084)	2.96 (0.171)	3.29 (0.122)	2.57 (0.305)	2.98 (0.208)	0.101
Localization	3.36 (0.084)	3.73 (0.156)	3.32 (0.122)	3.22 (0.348)	2.85 (0.202)	**0.012**
Margin achieved	3.56 (0.084)	3.65 (0.162)	3.53 (0.126)	3.72 (0.266)	3.39 (0.203)	0.745
Mitotic rate	3.57 (0.082)	3.81 (0.158)	3.55 (0.124)	2.83 (0.259)	3.56 (0.198)	0.053
Performance score	3.87 (0.079)	4.37 (0.122)	3.58 (0.118)	4.32 (0.276)	3.85 (0.210)	**0.000**
Presence of genetic prognostic maker(s)	2.94 (0.084)	2.79 (0.169)	3.06 (0.123)	2.78 (0.275)	2.93 (0.200)	0.559
Presence of (other) oncological diseases in history	2.95 (0.081)	3.11 (0.171)	2.87 (0.115)	3.06 (0.221)	2.78 (0.199)	0.534
Resectability	3.77 (0.078)	4.00 (0.154)	3.68 (0.115)	3.94 (0.249)	3.51 (0.204)	0.193
Size	4.20 (0.068)	4.57 (0.114)	4.10 (0.098)	4.58 (0.221)	3.62 (0.190)	**0.000**
Tumor differentiation	3.97 (0.074)	3.89 (0.170)	4.01 (0.103)	3.74 (0.295)	4.09 (0.155)	0.671

*Global *p* value for difference in distribution among specialties.
